# [Retracted] Downmodulation of dimethyl transferase activity enhances tumor necrosis factor-related apoptosis-inducing ligand-induced apoptosis in prostate cancer cells

**DOI:** 10.3892/ijo.2026.5926

**Published:** 2026-08-10

**Authors:** Claudio Festuccia, Giovanni Luca Gravina, Anna Maria D'Alessandro, Danilo Millimaggi, Cristiana Di Rocco, Vincenza Dolo, Enrico Ricevuto, Carlo Vicentini, Mauro Bologna

Int J Oncol 33: 381-388, 2008; DOI: 10.3892/ijo_00000019

Following the publication of this paper, it was drawn to the Editor's attention by a concerned reader that, regarding the western blot data, this paper appeared to contain a number of data duplications/erroneously assembled figures. First, one of the p-Erk1/2 bands in Fig. 2B (the leftmost lane) appeared to match one of the protein bands shown for Erk1/2 experiments (the rightmost lane). Secondly, Bcl-Xa bands in Fig. 2A were apparently matching with cIAP-1 bands in Fig. 5B (albeit with horizontal flipping and vertical resizing of the data). Thirdly, the cIAP-1 protein bands in Fig. 5B also appeared to have been re-used in a subsequent paper published by the same research group in the journal *Endocrine-Related Cancer*.

Upon performing an independent analysis of the data in this paper, a pair of further data duplications were identified; namely, phospho-p53 bands in Fig. 2A were apparently matching with Dr4 bands in Fig. 5B, and there also appeared to be an overlap of β-actin bands comparing Fig. 2B with Fig. 5B (where the reported experimental conditions were different). Given the apparent duplications of data within this paper itself and the re-use of data in a subsequently published paper, the Editor of *International Journal of Oncology* has decided that it should be retracted from the Journal on account of a lack of confidence in the presented data. The authors were asked for an explanation to account for these concerns, but the Editorial Office did not receive a reply. The Editor apologizes to the readership for any inconvenience caused.

